# Evaluation of Synergy Extrapolation for Predicting Unmeasured Muscle Excitations from Measured Muscle Synergies

**DOI:** 10.3389/fncom.2020.588943

**Published:** 2020-12-04

**Authors:** Di Ao, Mohammad S. Shourijeh, Carolynn Patten, Benjamin J. Fregly

**Affiliations:** ^1^Rice Computational Neuromechanics Lab, Department of Mechanical Engineering, Rice University, Houston, TX, United States; ^2^Biomechanics, Rehabilitation, and Integrative Neuroscience (BRaIN) Lab, VA Northern California Health Care System, Martinez, CA, United States; ^3^Department of Physical Medicine and Rehabilitation, Davis School of Medicine, University of California, Sacramento, CA, United States

**Keywords:** muscle synergy, EMG-driven modeling, stroke, principal component analysis (PCA), non-negative matrix factorization (NMF), muscle excitation, EMG normalization

## Abstract

Electromyography (EMG)-driven musculoskeletal modeling relies on high-quality measurements of muscle electrical activity to estimate muscle forces. However, a critical challenge for practical deployment of this approach is missing EMG data from muscles that contribute substantially to joint moments. This situation may arise due to either the inability to measure deep muscles with surface electrodes or the lack of a sufficient number of EMG channels. Muscle synergy analysis (MSA) is a dimensionality reduction approach that decomposes a large number of muscle excitations into a small number of time-varying synergy excitations along with time-invariant synergy weights that define the contribution of each synergy excitation to all muscle excitations. This study evaluates how well missing muscle excitations can be predicted using synergy excitations extracted from muscles with available EMG data (henceforth called “synergy extrapolation” or SynX). The method was evaluated using a gait data set collected from a stroke survivor walking on an instrumented treadmill at self-selected and fastest-comfortable speeds. The evaluation process started with full calibration of a lower-body EMG-driven model using 16 measured EMG channels (collected using surface and fine wire electrodes) per leg. One fine wire EMG channel (either iliopsoas or adductor longus) was then treated as unmeasured. The synergy weights associated with the unmeasured muscle excitation were predicted by solving a nonlinear optimization problem where the errors between inverse dynamics and EMG-driven joint moments were minimized. The prediction process was performed for different synergy analysis algorithms (principal component analysis and non-negative matrix factorization), EMG normalization methods, and numbers of synergies. SynX performance was most influenced by the choice of synergy analysis algorithm and number of synergies. Principal component analysis with five or six synergies consistently predicted unmeasured muscle excitations the most accurately and with the greatest robustness to EMG normalization method. Furthermore, the associated joint moment matching accuracy was comparable to that produced by initial EMG-driven model calibration using all 16 EMG channels per leg. SynX may facilitate the assessment of human neuromuscular control and biomechanics when important EMG signals are missing.

## Introduction

Knowledge of muscle forces could provide valuable insight into not only the neural control strategies employed by the central nervous system (CNS) (Contessa and Luca, 2013; Del Vecchio et al., 2018) but also the development of effective treatments for neuromusculoskeletal disorders (Shao et al., 2009; Fregly et al., 2012b, Fregly et al., 2012a; Allen et al., 2013; Pitto et al., 2019; Sauder et al., 2019). Since direct measurement of muscle force is generally not possible, computational techniques have been developed to generate muscle force estimates (Anderson and Pandy, 2001; Lloyd and Besier, 2003; Thelen et al., 2003; Buchanan et al., 2005; Shao et al., 2009). However, since the human musculoskeletal system possesses more muscles than degrees-of-freedom (DOFs) in the skeleton (i.e., the muscle redundancy problem), no unique muscle force solution exists unless either muscle activity patterns are defined by measured EMG signals (Lloyd and Besier, 2003; Manal and Buchanan, 2003; Buchanan et al., 2005; Shao et al., 2009; Kumar et al., 2012; Sartori et al., 2012; Meyer et al., 2017) or assumptions are made about how muscles contribute to the joint moments (e.g., energetic cost is minimized; Anderson and Pandy, 2001; Ackermann and van den Bogert, 2010; Shourijeh and McPhee, 2014). EMG-driven musculoskeletal modeling is a computational approach for predicting muscle forces that can bypass the muscle redundancy problem while simultaneously allowing for calibration of musculotendon properties (e.g., optimal muscle fiber length; Lloyd and Besier, 2003; Amarantini and Martin, 2004; Shao et al., 2009; Sartori et al., 2012; Meyer et al., 2017). In EMG-driven models, processed EMG and muscle-tendon kinematic data are input to a muscle force generation model (typically a Hill-type model) to predict muscle forces and corresponding net joint moments. Nonlinear optimization is then used to calibrate musculotendon model parameters such that predicted net joint moments match inverse dynamic joint moments as closely as possible.

In EMG-driven models, the quality of the measured EMG signals affects the reliability of the estimated muscle forces. Surface EMG recording, which is non-invasive and easily applicable, has been the most popular method for measuring muscle electrical activity for biomechanical studies. However, intrinsic potential challenges exist with surface EMG data that may limit the accuracy of estimated muscle forces, such as noisy signals from crosstalk between adjacent muscles, movement artifacts, and challenges in attaining the true maximum muscle excitation for EMG normalization (Farina et al., 2002; Racinais et al., 2013; Sartori et al., 2014). Beyond these issues, the inability to acquire EMG data from deep muscles that contribute substantially to joint moments is a practical challenge (Sartori et al., 2014; Zonnino and Sergi, 2019). For instance, it is practically impossible to collect EMG data from deep hip muscles (e.g., iliacus and psoas) using surface electrodes. However, when EMG data from important deep muscles are missing in an EMG-driven model, force estimates for other muscles that have similar roles may be significantly overestimated (Zonnino and Sergi, 2019). Compared to surface electrodes, fine wire electrodes are able to measure the electrical activity of deep muscles with lower levels of crosstalk (Péter et al., 2019). However, the use of fine wire electrodes requires special skills and longer set-up time and may cause discomfort and pain for the subject. Furthermore, in some cases, such as patients who have a cancerous tumor near an important deep muscle, use of a fine wire electrode may be contraindicated for safety reasons. Regardless of the EMG measurement technique, EMG-driven models require EMG data collection from a large number of muscles, which may not be possible due to EMG system limitations. Therefore, a computational method that can reliably estimate muscle excitations associated with missing EMG signals would be valuable for development of EMG-driven models.

Previous studies have explored computational methods for predicting unmeasured muscle excitations within EMG-driven models. Static optimization (SO) (Crowninshield and Brand, 1981; Anderson and Pandy, 2001; Damsgaard et al., 2006; Heintz and Gutierrez-Farewik, 2007; Pizzolato et al., 2015) has been embedded into the EMG-driven model calibration process to estimate missing muscle excitations. The objective function for this approach minimizes both joint moment tracking errors and activation levels associated with unmeasured muscle excitations (Sartori et al., 2014; Zonnino and Sergi, 2019). Zonnino et al. presented an EMG-driven forward dynamics estimator that used an SO-based neural model to determine unmeasured muscle activations. The approach reduced muscle force estimation error compared to a conventional estimator that neglected the contribution of unmeasured muscles (Zonnino and Sergi, 2019). Similarly, Satori et al. developed a hybrid EMG-informed model in which experimental EMG signals were minimally adjusted while missing EMG signals (e.g., from iliacus and psoas) were predicted via SO. However, none of these studies have provided evidence that predictions of unmeasured muscle activations were reliable and in reasonable agreement with experimental measurements. Furthermore, because time histories were not taken into account in SO, the resulting muscle activations might contain unrealistic discontinuities due to the optimization problem being solved one time frame at a time.

Another approach for estimating unmeasured muscle excitations is to use muscle synergy concepts. A muscle synergy is composed of a time-varying synergy excitation and a corresponding time-invariant synergy vector containing weights that define how each synergy excitation contributes to the excitation of all muscles (Tresch et al., 1999; Ting and Chvatal, 2010; Banks et al., 2017; Shourijeh and Fregly, 2020). While muscle synergies have been broadly used in descriptive research to analyze experimental muscle excitations during a large number of movement tasks (Ivanenko et al., 2005; Torres-Oviedo and Ting, 2007; Bowden et al., 2010; Walter et al., 2014; Kristiansen et al., 2015; Meyer et al., 2016; Ruiz Garate et al., 2017; Sauder et al., 2019), few studies have performed predictive analyses using muscle synergy information (Ajiboye and Weir, 2009; Meyer et al., 2016; Bianco et al., 2018; Sauder et al., 2019). Ajiboye and Weir demonstrated that subject-specific synergies extracted from muscle activities recorded for a subset of postures can be used to predict EMG patterns for the remaining postures. Bianco et al. investigated the theoretical feasibility of using synergy excitations extracted from a group of eight “included” muscle excitations treated as measured to construct muscle excitations for a group of eight “excluded” muscle excitations treated as unmeasured. However, the synergy vector weights associated with “remaining” postures in Ajiboye and Weir (2009) or “excluded” muscles in Bianco et al. (2018) were not predicted without knowledge of the muscle excitations being treated as unmeasured but rather were fitted with knowledge of those excitations using least square algorithms. Several other studies have imposed synergy structures on muscle excitations or activations through optimization when estimating knee contact force (Walter et al., 2014), joint stiffness (Shourijeh and Fregly, 2020), or motion (Clark et al., 2009; Allen and Neptune, 2012; Meyer et al., 2016; Mehrabi et al., 2019; Falisse et al., 2020). Imposing a muscle synergy structure on predicted muscle excitations or activations can not only eliminate discontinuities between neighboring time frames but also reduce the number of design variables in the optimization problem. These benefits occur when the estimated synergy vector weights are treated as invariant across time frames, necessitating that the optimization problem be solved over all time frames simultaneously. However, to the best of the authors' knowledge, the reliability with which unmeasured muscle excitations can be estimated using EMG-driven models with unknown synergy vector weights has not been studied previously.

This study evaluated how well a synergy-based muscle excitation estimation method involving EMG-driven modeling, termed “synergy extrapolation” or “SynX,” is able to predict muscle excitations that cannot be measured experimentally. Since the outcome of muscle synergy analysis is affected by methodological choices, such as EMG processing (e.g., magnitude normalization method), physiological assumptions (e.g., number of synergies), and matrix decomposition algorithm [e.g., principal component analysis (PCA) or non-negative matrix factorization (NMF)] (Tresch et al., 2006; Hug et al., 2012; Steele et al., 2013; Oliveira et al., 2014; Banks et al., 2017; Shuman et al., 2017; Ebied et al., 2018; Gallina et al., 2018), we also evaluated how these choices affect SynX results. The evaluation was performed using a gait data set collected from a high-functioning subject post-stroke performing treadmill walking at self-selected and fastest-comfortable speeds. One muscle excitation measured using a fine wire EMG electrode (i.e., from either iliopsoas or adductor longus) was treated as missing, and SynX was applied to an EMG-driven model with calibrated musculotendon parameters to predict the missing muscle excitation. By quantitatively evaluating the differences in SynX performance produced by different methodological choices, this work provides evidence-based suggestions for which methods are likely to produce the most accurate predictions of missing muscle excitations.

## Materials and Methods

### Experimental Data

A previously published gait data set collected from one high-functioning stroke survivor (age 79 years, LE Fugl-Meyer Motor Assessment 32/34 pts, right-sided hemiparesis, height 1.7 m, mass 80.5 kg) was used to evaluate the SynX process (Meyer et al., 2017). Motion capture (100 Hz, Vicon Corp., Oxford, UK), ground reaction force (1000 Hz, Bertec Corp., Columbus, OH), and EMG (1000 Hz, Motion Lab Systems, Baton Rouge, LA) data were recorded simultaneously while the subject walked on a split-belt instrumented treadmill (Bertec Corp., Columbus, OH) at two speeds: 0.5 m/s (self-selected speed) and 0.8 m/s (fastest-comfortable speed). All experimental procedures were approved by the University of Florida Health Science Center Institutional Review Board (IRB-01), and the subject provided written informed consent before participation. Motion capture and ground reaction force (GRF) data were low-pass filtered using a fourth-order zero-phase lag Butterworth filter with a cut-off frequency at 7/*t*_*f*_ Hz, where *t*_*f*_ is the period of the gait cycle being processed (McLean et al., 2005; Meyer et al., 2017). Sixteen channels of EMG data were collected from each leg using 11 surface and 5 fine wire electrodes, which made EMG data available from important deep muscle groups (e.g., iliopsoas) (Supplementary Table 1). After being high-pass filtered at 40 Hz, demeaned, full-wave rectified, and low-pass filtered at 3.5/*t*_*f*_ Hz, each EMG signal was normalized to the maximum value over all trials. After processing, each trial of EMG data was time-normalized by resampling to 101 time frames per gait cycle (heel strike to heel strike) using cubic spline data interpolation. See Meyer et al. (2017) for further details.

### Musculoskeletal Model

A generic full-body OpenSim musculoskeletal model (Arnold et al., 2010) was adopted for analyses in OpenSim v3.3 (Delp et al., 2007; Seth et al., 2018). The model controlled 5 degrees of freedom (DOFs) including two hip DOFs [flexion/extension (HipFE) and adduction/abduction (HipAA)], one knee DOF [flexion/extension (kneeFE)], and two ankle DOFs [plantarflexion/dorsiflexion (AnklePD) and inversion/eversion (AnkleIE)]. Of the 45 muscles in each leg present in the original model, 35 muscles per leg were retained. Compartments of muscles with similar anatomic function (e.g., semimembranosus and semitendinosus) shared a common EMG signal (Meyer et al., 2017). To personalize the model, we performed five steps sequentially on the modified generic OpenSim model using a combination of OpenSim and custom Matlab analyses: (1) model scaling to match the subject's anthropometry; (2) kinematic calibration to determine personalized lower body joint positions and orientations (Reinbolt et al., 2005); (3) inverse kinematics (IK) to calculate joint angle time histories from the surface marker data using the calibrated kinematic model; (4) surrogate musculoskeletal geometry creation to fit muscle-tendon lengths and moment arms as polynomial functions of lower body joint angles and velocities (Menegaldo et al., 2004; Meyer et al., 2017); and (5) inverse dynamics (ID) to obtain experimental joint moments using experimental GRF data and IK-derived time histories of joint kinematics as inputs.

### EMG-Driven Musculoskeletal Model

To predict muscle forces and net joint moments in the lower extremities with processed EMG data, a previously developed and published EMG-driven model was employed (Meyer et al., 2017), where muscles were treated as Hill-type models (Hill, 1938; Zajac, 1989) with a rigid tendon. The joint moment produced by a muscle spanning a particular joint can be represented as:

(1) $$\begin{array}{l} M=r\cdot F_{o}^{M}\cdot \left[a\cdot f_{l}\left({\tilde{l}}^{M}\left(t\right)\right)\cdot f_{v}\left({\tilde{v}}^{M}\left(t\right)\right)+f_{p}\left({\tilde{l}}^{M}\left(t\right)\right)\right]\text{cos }\alpha \end{array}$$

where *M* is the moment generated by the muscle about the joint, *r* is the moment arm of the muscle about the same joint, $F_{o}^{M}$ is the maximum isometric force of the muscle, *a* is the muscle activation, ${\tilde{l}}^{M}\left(t\right)$ and $\tilde{v}$^*M*^(*t*) are the time-varying normalized muscle fiber length and velocity, respectively, and α is the pennation angle of the muscle. $f_{l}\left({\tilde{l}}^{M}\left(t\right)\right)$ and $f_{v}\left({\tilde{v}}^{M}\left(t\right)\right)$ define the normalized muscle active force-length and active force-velocity relationships, while $f_{p}\left({\tilde{l}}^{M}\left(t\right)\right)$ defines the normalized muscle passive force-length relationship (Zajac, 1989; Meyer et al., 2017).

Muscle activation (*a*) was calculated from muscle excitation (*e*) using a published model of muscle activation dynamics (He et al., 1991; Lloyd and Besier, 2003; Meyer et al., 2017). The model uses a first-order differential equation (Equation 2) to define the muscle excitation (*e*) to neural activation (μ*)* relationship and a nonlinear function (Equation 3) to define the neural activation (μ*)* to muscle activation (*a*) relationship:

(2) $$\begin{array}{l} \frac{du\left(t\right)}{dt}=\left(c_{1}e\left(t-d\right)+c_{2}\right)\left(e\left(t-d\right)-u\left(t\right)\right) \end{array}$$

(3) $$\begin{array}{l} a\left(t\right)=\left(1-c_{3}\right)u\left(t\right)+c_{3}\left[\frac{g_{1}}{{g_{2}\left(u\left(t\right)+g_{3}\right)}^{g_{4}}+g_{5}}+1\right] \end{array}$$

where $c_{1}=\;\frac{1}{\tau_{act}}-\;\frac{1}{\tau_{dact}}$, $c_{2}=\;\frac{1}{\tau_{dact}}$, and *c*_3_ is an activation nonlinearity constant. For each muscle, τ_*act*_ and τ_*dact*_ are activation and deactivation time constants, respectively, and τ_*dact*_ is assumed to be 4τ_*act*_ (Zajac, 1989; Meyer et al., 2017). *d* denotes an electromechanical time delay. *g*_1_ to *g*_5_ are constant coefficients that were determined by fitting published experimental data from isometric contractions (Manal and Buchanan, 2003). Muscle excitations were derived by multiplying the processed EMG signals by muscle-specific scale factors between 0.05 and 1 to reflect unknown maximum excitation levels.

SynX was evaluated by following a two-step procedure. The first step calibrated an EMG-driven model of each leg using a full set of 16 EMG channels per leg (henceforth called “full EMG-driven”). For this model, every muscle was associated to an experimentally measured EMG signal collected using surface or fine wire electrodes. To perform full EMG-driven model calibration, we used experimental walking data over 10 gait cycles from the two walking speeds (five trials per speed) (henceforth called “calibration trials”). During this step, a sequence of optimizations was performed to identify the parameter values required by the activation dynamics model, Hill-type muscle-tendon model, and surrogate musculoskeletal geometric model (described in Meyer et al., 2017) that reproduced the lower-body inverse dynamic joint moments as closely as possible (Equation 4). The primary cost function for EMG-driven calibration was formulated as:

(4) $$\begin{array}{l} J≜\overset{N}{\underset{i=1}{\sum }}{\left(M_{i}^{mod}-M_{i}^{\text{exp}}\right)}^{2} \end{array}$$

where $M_{i}^{mod}$ is the model-predicted moment about joint *i*, $M_{i}^{\text{exp}}$ is the experimental moment about joint *i* calculated using inverse dynamics, and *N* is the total number of joints. The model parameter values calibrated for each muscle-tendon actuator by the optimization process included: electromechanical delay, activation time constant, activation nonlinearity constant, EMG scale factor, optimal muscle fiber length, tendon slack length, and geometric coefficients defining muscle-tendon lengths, velocities, and moment arms. Further details on the specification of initial guesses, variable bounds, overall cost function structure, additional constraints, and penalty terms for calibrating the EMG-driven model can be found in Meyer et al. (2017). The calibrated model was used in subsequent steps of the SynX evaluation process.

### Synergy Extrapolation Methodology

**Figure 1:**
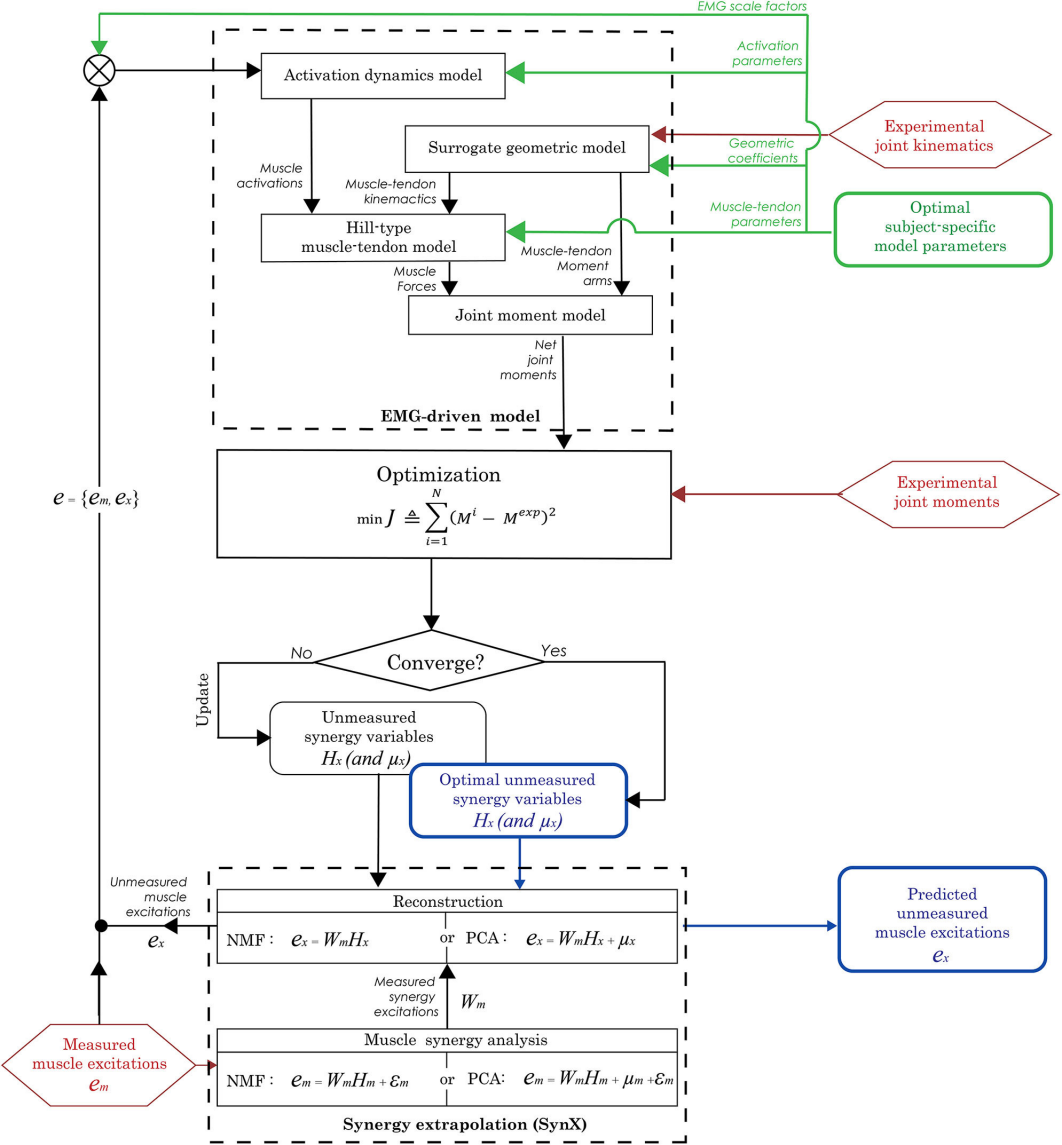
Flowchart of the synergy extrapolation (SynX) process using an EMG-driven model. Prior to performing SynX, we calibrated musculotendon model parameter values in the EMG-driven model (green color) using a full set of 16 EMG signals per leg, collected using surface and fine wire electrodes. Then one fine wire EMG signal (either iliopsoas or adductor longus) was treated as unmeasured and predicted using SynX. The unknown synergy vector weights *H*_*x*_ in both PCA and NMF and offsets μ_*x*_ in PCA for the unmeasured muscle excitation were predicted by solving a non-linear optimization problem where the errors between inverse dynamics and EMG-driven joint moments were minimized while all musculotendon model parameters were held constant at the calibrated values.

The second step in the evaluation process predicted unmeasured muscle excitations using SynX within the EMG-driven model calibrated in the first step (Figure 1). Specifically, the EMG signals from hip muscles that were recorded using fine wire electrodes were removed one at a time (either iliopsoas or adductor longus) and treated as unmeasured, while the remaining 15 channels of EMG data were treated as measured. We performed muscle synergy analysis on a trial by trial basis for all measured muscle excitations (*e*_*m*_) to extract a low-dimensional set of time-varying measured synergy excitations (*W*_*m*_) and a corresponding time-invariant synergy vector containing weights (*H*_*m*_) that defined how each synergy excitation contributed to the excitation of the measured muscles. Next, unmeasured muscle excitations (*e*_*x*_) were constructed using the measured synergy excitations (*W*_*m*_) along with a trial-specific time-invariant synergy vector containing weights (*H*_*x*_) associated with the unmeasured muscle. During this step, EMG-driven joint moments were estimated using a combination of both measured and unmeasured muscle excitations (*e* = {*e*_*m*_, *e*_*x*_}), and the unmeasured synergy vector weights (*H*_*x*_) were identified iteratively through optimization by tracking experimental inverse dynamics joint moments (Equation 4). For NMF, the unmeasured synergy vector weights (*H*_*x*_) were given a lower bound of zero, while they were unbounded for PCA. The unmeasured synergy vector weights (*H*_*x*_) were initialized by the optimization using randomly chosen values between 0 and 1. The unmeasured muscle excitations predicted by SynX were constrained to be between 0 and 1.

We implemented the second step of the evaluation process on the 10 calibration trials used in the first step as well as 10 other trials not used for calibration (five trials per speed, henceforth called “evaluation trials”). Both steps of the evaluation process were performed with Matlab's built-in “fmincon” optimization algorithm using sequential quadratic programming. The structure of unmeasured synergy variables was slightly different between the matrix factorization algorithms, and more details are provided in the section below on methodological choices for synergy extrapolation.

### Methodological Choices for Synergy Extrapolation

Muscle synergy analysis requires a number of methodological choices that can influence the results of the analysis (Banks et al., 2017). Methodological choices that have been studied include EMG processing approaches (e.g., filtering parameters, normalization methods), assumptions about neural control complexity (e.g., number of synergies, number and choice of muscles, synergy vector variability across trials), matrix decomposition algorithm, and post-processing of results (Ivanenko et al., 2005; Tresch et al., 2006; Hug et al., 2012; Steele et al., 2013; Oliveira et al., 2014; Shourijeh et al., 2016; Banks et al., 2017; Shuman et al., 2017; Ebied et al., 2018; Gallina et al., 2018; Mehryar et al., 2020). In the present study, for each subset of measured muscles, we performed SynX using a total of 80 methodological combinations comprised of two algorithms for matrix factorization, five methods for EMG normalization, and eight choices for number of muscle synergies (see Table 1 for summary).

**Table 1:** Methodological choices for synergy extrapolation.

**Description**	**Methods**	**Abbreviations**
Matrix factorization algorithm	Principal component analysis	PCA
	Non-negative matrix factorization	NMF
EMG normalization method	Maximum value over all trial	MaxOver
	Maximum value per trial	MaxPer
	Unit variance over all trials	VarOver
	Unit variance per trial	VarPer
	Unit magnitude per trial	MagPer
Number of muscle synergies	3, 4, 5, 6, 7, 8, 9, 10	

To calculate measured muscle synergies, we used the two matrix factorization algorithms that render the most divergent MSA results: non-negative matrix factorization (NMF) and principal component analysis (PCA) (Olree and Vaughan, 1995; Lee and Seung, 1999; d'Avella et al., 2003; Tresch et al., 2006; Ting and Chvatal, 2010; Banks et al., 2017; Bianco et al., 2018; Ebied et al., 2018). Both algorithms minimize the errors between the reconstructed and original data sets. NMF uses nonlinear optimization to find a potentially non-unique solution iteratively subject to constraints on non-negativity, where non-uniqueness is the result of the non-convexity of the search space (Shourijeh et al., 2016). In contrast, PCA uses linear algebra to find a unique solution analytically subject to constraints on orthogonality but not non-negativity. PCA identifies the internal structure of the data that best explains its variance (Torres-Oviedo and Ting, 2007; Ting and Chvatal, 2010). During SynX with a given number of synergies, muscle excitations in the measured subset of 15 muscles (*e*_*m*_) were represented as:

(5) $$\begin{array}{l} e_{m}=\{\begin{array}{cc} W_{m}H_{m}+\varepsilon_{m} & \left(NMF\right)\\ W_{m}H_{m}+{\mu_{m}+\varepsilon }_{m} & \left(PCA\right) \end{array} \end{array}$$

where *e*_*m*_ denotes an *n* time points × 15 measured EMG signals matrix that contains measured muscle excitations in columns, *W*_*m*_ denotes an *n* time points × *p* synergies matrix that contains measured synergy excitations in columns, and *H*_*m*_ denotes a *p* synergies × 15 measured EMG signals matrix that contains measured synergy vector weights. In preparation for NMF or PCA, measured EMG signals from each gait cycle were re-sampled to 101 time frames plus 10/*t*_*f*_ time frames before the start of the cycle to account for a maximum electromechanical delay of 100 ms. For NMF and PCA, ε_*m*_ denotes the part of *e*_*m*_ that cannot be explained by *W*_*m*_*H*_*m*_, while for PCA, μ_*m*_ specifies the average muscle excitations in *e*_*m*_. Matlab functions “nnmf” (alternating least squares algorithm with 10 replicates) and “pca” were used to perform NMF and PCA, respectively.

Following MSA by either approach, the unmeasured muscle excitation (*e*_*x*_) (either iliopsoas or adductor longus) was constructed from the measured synergy excitations (*W*_*m*_) using the following relationships:

(6) $$\begin{array}{l} e_{x}=\{\begin{array}{cc} W_{m}H_{x} & \left(NMF\right)\\ W_{m}H_{x}+\mu_{x} & \left(PCA\right) \end{array} \end{array}$$

where *H*_*x*_ is a *p*-synergy × 1 vector representing the unmeasured muscle synergy vector weights. Unlike NMF, PCA needs an additional design variable μ_*x*_ that represents the average value of each unmeasured muscle excitation. Both *H*_*x*_ and μ_**x**_ were calibrated by tracking experimental joint moments within our EMG-driven modeling framework (Equation 4) while keeping all musculotendon model parameters at their calibrated values.

Because EMG normalization method affects MSA results, this study explored five approaches for normalizing the magnitudes of processed EMG signals in preparation for MSA. EMG normalization was performed either within individual trials (Per trial) or across all trials (Over all trials). Specifically, each processed muscle EMG signal was normalized using either: (1) maximum value over all trials (MaxOver), (2) maximum value per trial (MaxPer), (3) unit variance over all trials (VarOver), (4) unit variance per trial (VarPer), and (5) unit magnitude per trial (MagPer) (Banks et al., 2017). VarOver and VarPer normalizations involved dividing each processed EMG signal by its standard deviation over all trials and in each trial, respectively. MagPer normalization involved dividing each EMG signal by its 2-norm value for each trial (Banks et al., 2017).

Since the specified number of muscle synergies also affects the outcome of MSA, we repeated the SynX process for three through 10 synergies. This range was chosen since three to six muscle synergies have been shown to be sufficient to account for over 90% of the variability in up to 30 muscle excitations during human movement (Ivanenko et al., 2005; Ting and Macpherson, 2005; Cappellini et al., 2006; Bianco et al., 2018). Similarity in both magnitude and shape between SynX-predicted and experimental excitations for unmeasured muscles was taken into account when determining the optimal number for synergies (see next section for details).

### Evaluation Metrics

Several common metrics were employed to score outcomes of muscle synergy analysis, performance of SynX, and accuracy of joint moment estimates with different combinations of methodological choices. Variance accounted for (VAF) was calculated to compare the ability of different methodological combinations to reconstruct measured muscle excitations (Tresch et al., 2006; Steele et al., 2013; Shourijeh et al., 2016; Banks et al., 2017). Root mean square error (RMSE) and Pearson correlation coefficient *r* between experimental and model-predicted unmeasured muscle excitations across all trials were computed to quantitatively assess matching of magnitude and shape, respectively. In addition, RMSE and *r*-values were calculated for each trial, and the frequency with which the number of synergies possessing the highest *r*-values or lowest RMSE values appeared was analyzed. The correlation between predicted and experimental unmeasured muscle excitations was interpreted quantitatively as weak (*r* < 0.35), moderate (0.35 < *r* ≤ 0.67), strong (0.67 < *r* ≤ 0.9), or very strong (*r* ≥ 0.9) (Taylor, 1990). Mean absolute errors (MAE) between experimental and model-predicted joint moments were calculated across all gait cycles to evaluate the accuracy of the predicted joint moments during full EMG-driven calibration (step 1) and the SynX process (step 2).

### Statistical Analyses

Multiple statistical analyses were performed to assess whether the calculated metrics resulting from different SynX methodological choices were statistically different for each unmeasured muscle-leg combination. First, to assess whether reconstruction performance of measured muscle excitations was statistically different between the two matrix factorization algorithms and the five EMG normalization methods, we performed a two-factor ANOVA with a Tukey-Kramer *post-hoc* analysis on VAF values. Second, to compare SynX performance for different methodological choices, we performed two three-factor (matrix factorization algorithm by EMG normalization method by number of synergies) ANOVA tests on *r* and RMSE values between predicted and experimental unmeasured muscle excitations across all calibration and evaluation trials, respectively. In addition, we performed paired *t*-tests on *r* and RMSE values to investigate whether matrix factorization algorithm (i.e., PCA and NMF) had a significant influence on SynX performance for the same number of synergies. Third, we performed a three-factor (matrix factorization algorithm by EMG normalization method by number of synergies) ANOVA to compare MAE values characterizing the accuracy of joint moment tracking from different approaches. All statistical analyses were performed in Matlab, and significance levels were set at *p* < 0.05.

## Results

### Muscle Synergy Analysis

The two-way ANOVA for mean VAF values revealed main effects of matrix factorization algorithm (*p* < 0.01) and EMG normalization method (*p* < 0.01) on the variance explained by factorization of measured muscle excitations. For five or fewer synergies, PCA generally had significantly higher VAF values than did NMF for the same number of synergies (all *p* < 0.05, gray shading in Table 2). Overall, extracted synergy excitations were able to predict the measured muscle excitations with > 90% VAF using three or more synergies in the left leg and four or more synergies in the right leg for PCA and 4 or more synergies in both legs for NMF (Table 2). Across all EMG normalization methods, MaxOver produced significantly higher VAF values than did VarOver (*p* < 0.01), VarPer (*p* = 0.028), and MagPer (*p* < 0.01), while MaxPer produced the lowest VAF amongst the five EMG normalization methods (*p* < 0.01). Moreover, no statistically significant interaction was observed between matrix factorization algorithm and EMG normalization method for mean VAF values.

**Table 2:** Mean VAF values for PCA/NMF reconstruction of measured muscle excitations across all trials using three to seven synergies and 6 EMG normalization methods when either iliopsoas or adductor longus was assumed to be unmeasured.

**Unmeasured** **muscle**	**EMG** **normalization**	**Left leg (non-paretic)**					**Right leg (paretic)**				
		**Number of synergies**					**Number of synergies**				
		**3**	**4**	**5**	**6**	**7**	**3**	**4**	**5**	**6**	**7**
Iliopsoas	MaxOver	92.7/88.8	97.7/95.4	99.1/98.0	99.7/98.8	99.9/99.3	89.7/82.3	95.2/91.0	98.1/95.4	99.2/97.5	99.7/98.5
	MaxPer	90.8/86.5	97.2/94.7	98.8/97.5	99.5/98.5	99.8/99.1	85.6/77.7	93.1/86.8	97.2/93.1	98.7/96.3	99.5/97.6
	VarOver	90.1/86.4	97.4/94.4	98.9/97.6	99.6/98.5	99.9/99.0	87.8/77.8	94.6/89.1	97.8/94.1	99.1/96.4	99.7/97.6
	VarPer	90.9/87.3	97.2/94.6	98.8/97.5	99.5/98.5	99.8/99.1	85.7/77.9	93.3/86.6	97.2/92.7	98.7/96.2	99.5/97.8
	MagPer	92.5/88.5	97.4/95.0	98.9/97.7	99.6/98.6	99.9/99.2	83.5/75.6	91.9/85.9	96.7/91.5	98.8/95.6	99.5/97.6
Adductor	MaxOver	94.0/90.1	97.9/95.8	99.0/97.8	99.7/98.8	99.9/99.3	88.8/81.3	94.3/90.5	97.5/94.8	99.0/97.2	99.7/98.5
longus	MaxPer	91.9/87.8	97.2/94.7	98.7/97.2	99.5/98.4	99.8/99.0	85.1/77.2	91.7/86.4	96.5/92.6	98.4/95.7	99.5/97.5
	VarOver	93.2/87.6	97.5/94.6	98.8/97.4	99.6/98.4	99.9/99.0	86.1/78.0	93.2/88.6	96.8/93.3	98.6/95.5	99.6/97.5
	VarPer	92.4/88.3	97.3/94.7	98.7/97.2	99.5/98.5	99.8/99.0	84.7/76.5	92.1/86.1	96.5/92.1	98.5/95.8	99.5/97.6
	MagPer	92.0/87.7	96.9/94.3	98.6/97.1	99.5/98.2	99.8/98.8	83.0/74.4	90.8/85.1	96.3/90.4	98.4/95.1	99.5/96.7

*Gray shading represents a statistically significant difference (p ≤ 0.05) between PCA and NMF with matched EMG normalization method and matched number of synergies*.

### Synergy Extrapolation Performance

**Figure 2:**
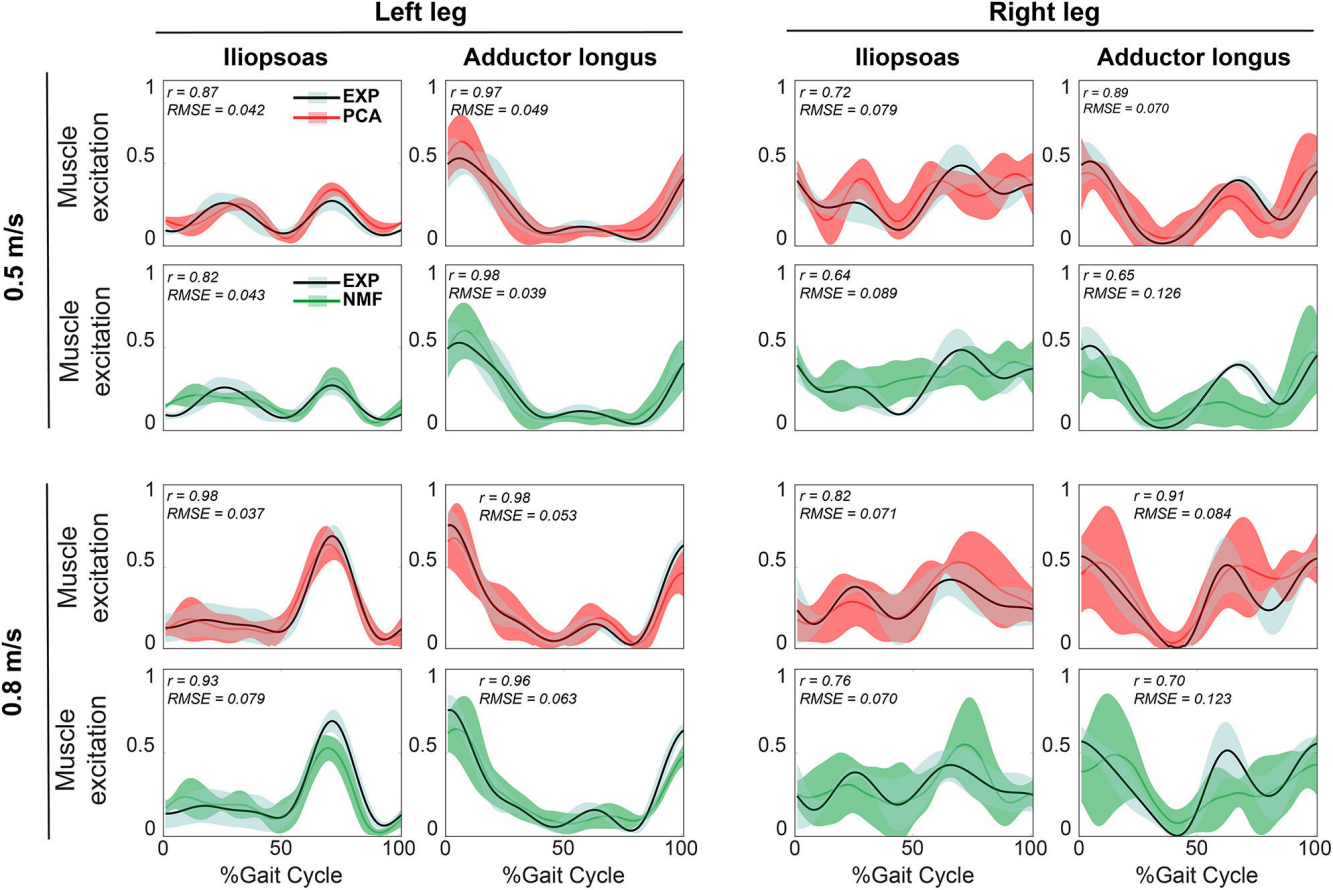
Representative results of reconstructed unmeasured muscle excitations across all calibration walking trials at the same speed using SynX (black line: average experimental curve; red line: PCA-based SynX; green line: NMF-based SynX; shaded area: ±1 standard deviation). Measured synergy excitations were calculated using the MaxOver EMG normalization method with six synergies. Results are reported over the complete gait cycle where 0% is heel strike and 100% is subsequent heel strike of the same leg (left leg: non-paretic, right leg: paretic). *r* and RMSE values were computed between average experimental and SynX-predicted muscle excitations.

**Figure 3:**
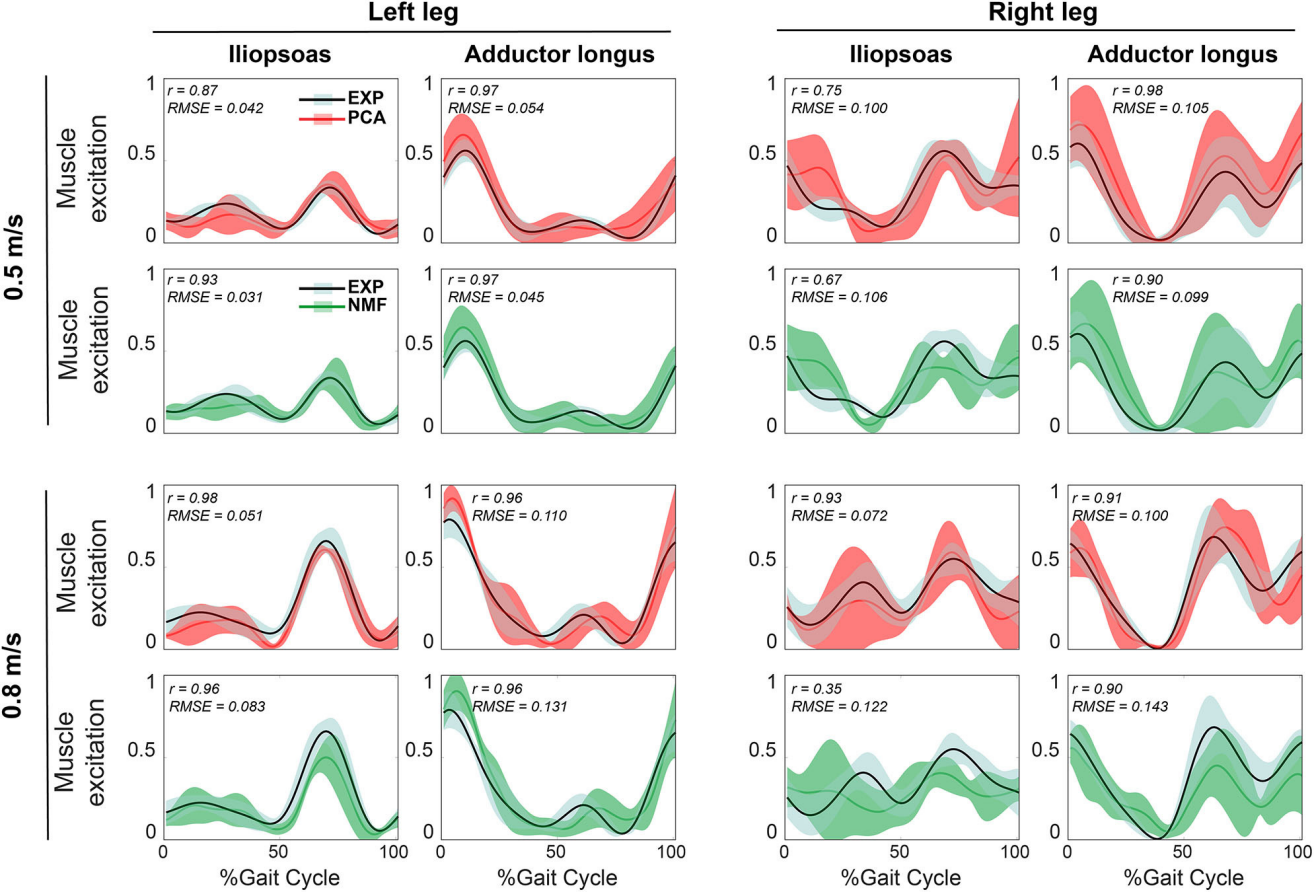
Representative results of average reconstructed unmeasured muscle excitations across all evaluation walking trials at the same speed using SynX (black line: average experimental curve; red line: PCA-based SynX; green line: NMF-based SynX; shaded area: ±1 standard deviation). Measured synergy excitations were calculated using the MaxOver EMG normalization method with six synergies. Results are reported over the complete gait cycle where 0% is heel strike and 100% is subsequent heel strike of the same leg (left leg: non-paretic, right leg: paretic). *r* and RMSE values were computed between average experimental and SynX-predicted muscle excitations.

For both calibration (Figure 2) and evaluation (Figure 3) trials, mean predicted unmeasured muscle excitations using PCA were strongly correlated with the corresponding experimental muscle excitations (mean *r* always ≥ 0.7), which was not consistently observed for the NMF results. Furthermore, RMSE values between the average predicted and actual unmeasured muscle excitations across all trials using PCA-based SynX were generally lower than those using NMF-based SynX. In addition, SynX performed using either PCA or NMF predicted more accurate unmeasured muscle excitations with less trial-to-trial variability for the left leg than for the right leg (Figures 2, 3).

**Figure 4:**
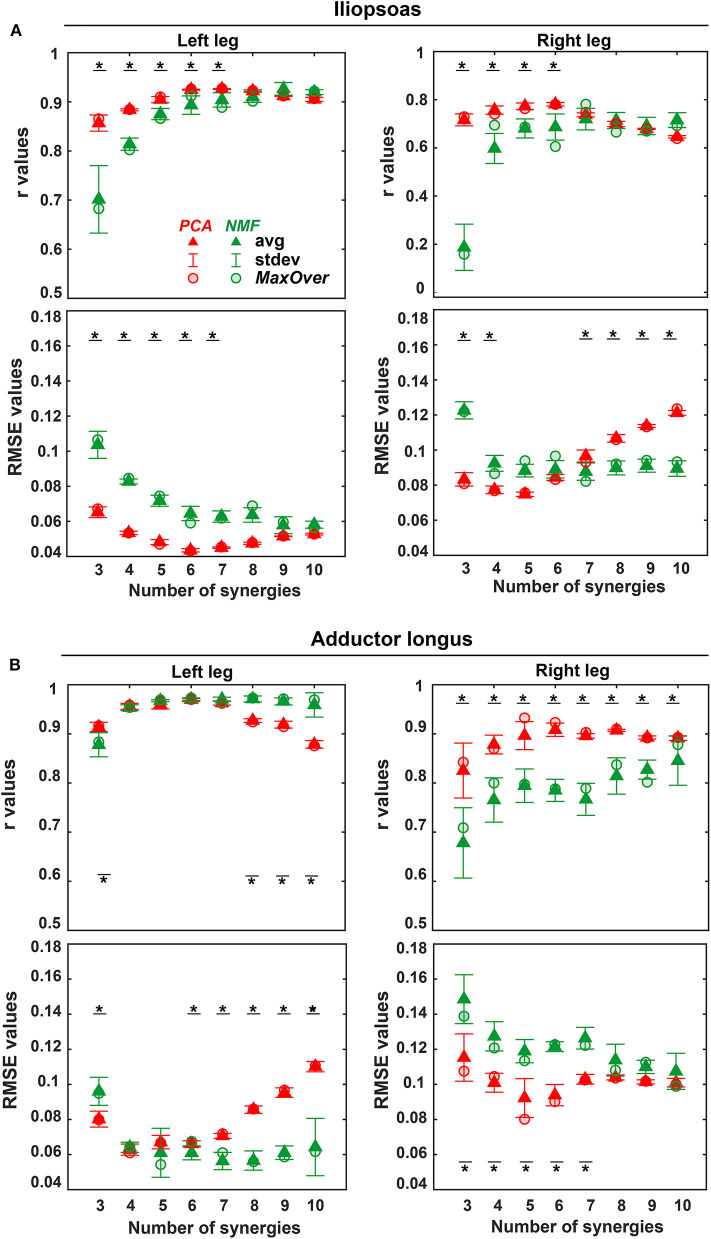
Average (triangles) and standard deviation of *r* and RMSE values for the reconstruction of iliopsoas **(A)** and adductor longus **(B)** muscle excitations across all trials (including both calibration trials and evaluation trials) and across all 5 EMG normalization methods for both legs (left leg: non-paretic, right leg: paretic) using three to 10 synergies (red: PCA-based SynX; green: NMF-based SynX. Red circular (PCA) and green circular (NMF) markers show average values across all trials using MaxOver normalization. A black bar with a star represents a statistically significant difference (*p* < 0.05) between PCA and NMF for the same number of synergies.

The three-factor ANOVA analyses revealed that the number of synergies (*p* < 0.01) and matrix factorization algorithm (*p* < 0.01) had a significant effect on both *r* and RMSE values for all unmeasured muscle-leg combinations, while EMG normalization method did not. Additionally, no statistically significant interaction was detected among the three factors for both *r* and RMSE values. For each unmeasured muscle-leg combination, as the number of synergies increased, PCA produced non-monotonic changes in *r* and RMSE values, with *r* values reaching a maximum and RMSE values a minimum at five or six synergies. Unlike PCA, *r* values for NMF initially rose with an increasing number of synergies and then remained high with further increases, while RMSE values initially dropped and then leveled off (Figure 4). Moreover, for the same number of synergies, PCA generally exhibited less variance than did NMF in mean *r* and RMSE values across the five EMG normalization methods (Figure 4 and Supplementary Figure 2).

**Figure 5:**
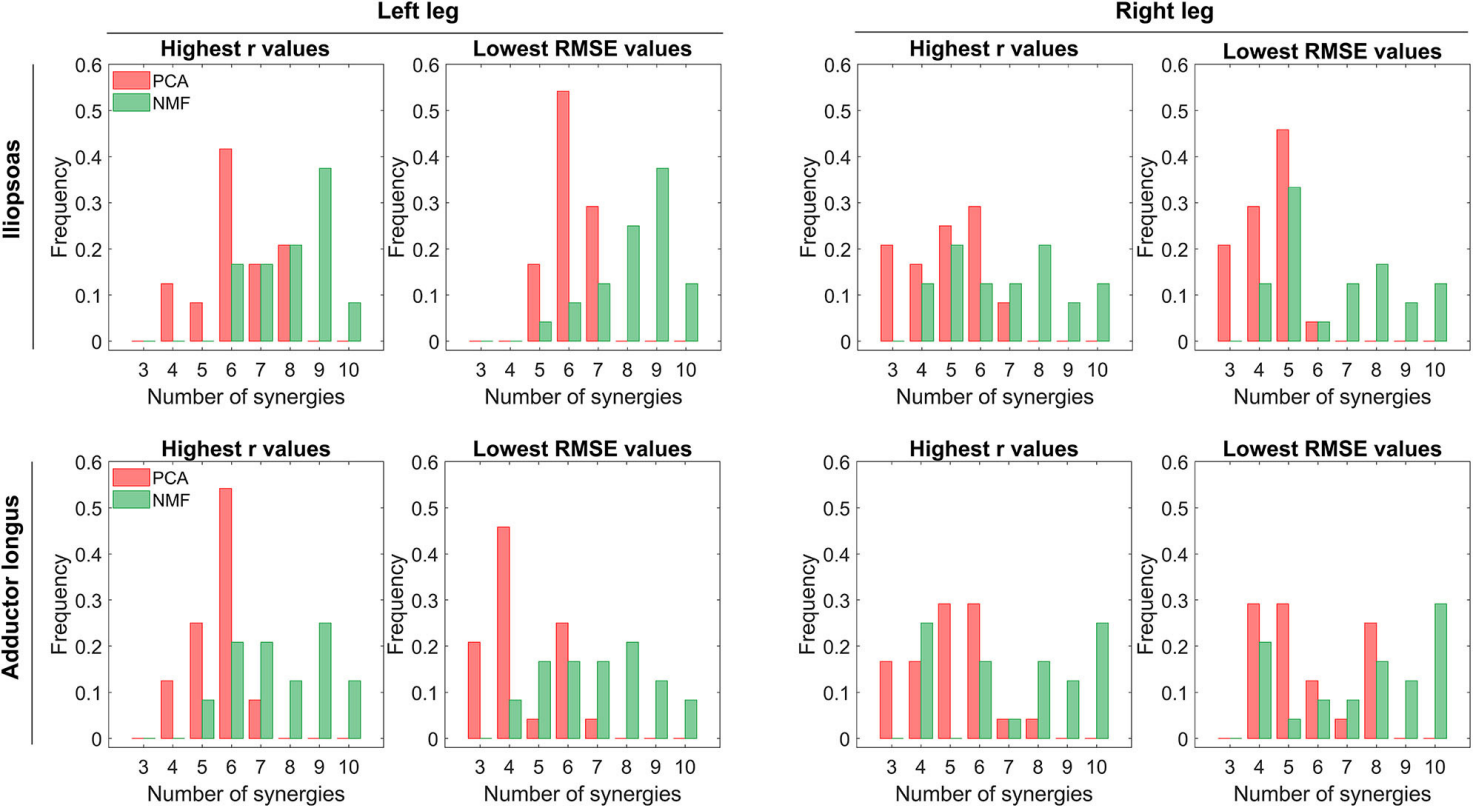
Distribution of the number of synergies that produce maximum *r* values or minimum RMSE values across all trials (including both calibration and evaluation) and all EMG normalization methods. In each histogram, the horizontal axis reports the number of synergies, and the vertical axis shows the frequency with which the number of synergies generates the best prediction of iliopsoas or adductor longus muscle excitation in terms of shape (indicated by *r*-values) and magnitude (indicated by RMSE-values). Red and green bars represent PCA-based and NMF-based synergy extrapolations, respectively. The left leg is non-paretic and the right leg is paretic.

PCA achieved significantly higher *r* values and lower RMSE values than did NMF when the number of synergies varied from 3 to 6 (*p* < 0.01). The one exception was adductor longus in the left leg, where the only significant difference between PCA and NMF occurred for 3 synergies (*p* < 0.01). When the number of synergies increased above 6, NMF provided substantially higher *r* values for adductor longus in the left leg (*p* < 0.05, Figure 4A), whereas PCA had markedly higher *r* values for adductor longus in the right leg (*p* < 0.01, Figure 4B). Additionally, for more than six synergies, RMSE values using NMF were significantly smaller than those found using PCA for iliopsoas in the right leg (*p* < 0.01, Figure 4A) and for adductor longus in the left leg (*p* < 0.01, Figure 4B). When similarity in both shape and magnitude were taken into account, based on average *r* and RMSE values, the best number of synergies for predicting unmeasured excitations using PCA was six for iliopsoas in the left leg (*r* = 0.93; RMSE = 0.043), five for iliopsoas in the right leg (*r* = 0.79; RMSE =0.073), five for adductors longus in the left leg (*r* = 0.97; RMSE =0.06), and five for adductors longus in the right leg (*r* = 0.93; RMSE = 0.081). Additionally, for most trials, PCA required fewer synergies (between 3 and 8) than did NMF (between 4 and 10) to achieve the best SynX performance (see histogram in Figure 5). For example, for iliopsoas in the left leg using five to eight synergies, 86% of trials could achieve the highest *r* values with PCA while only 54% could with NMF. Similarly, with five to eight synergies, the smallest RMSE values could be obtained for 100% of trials using PCA but only 50% of trials using NMF.

### Joint Moment Prediction

**Figure 6:**
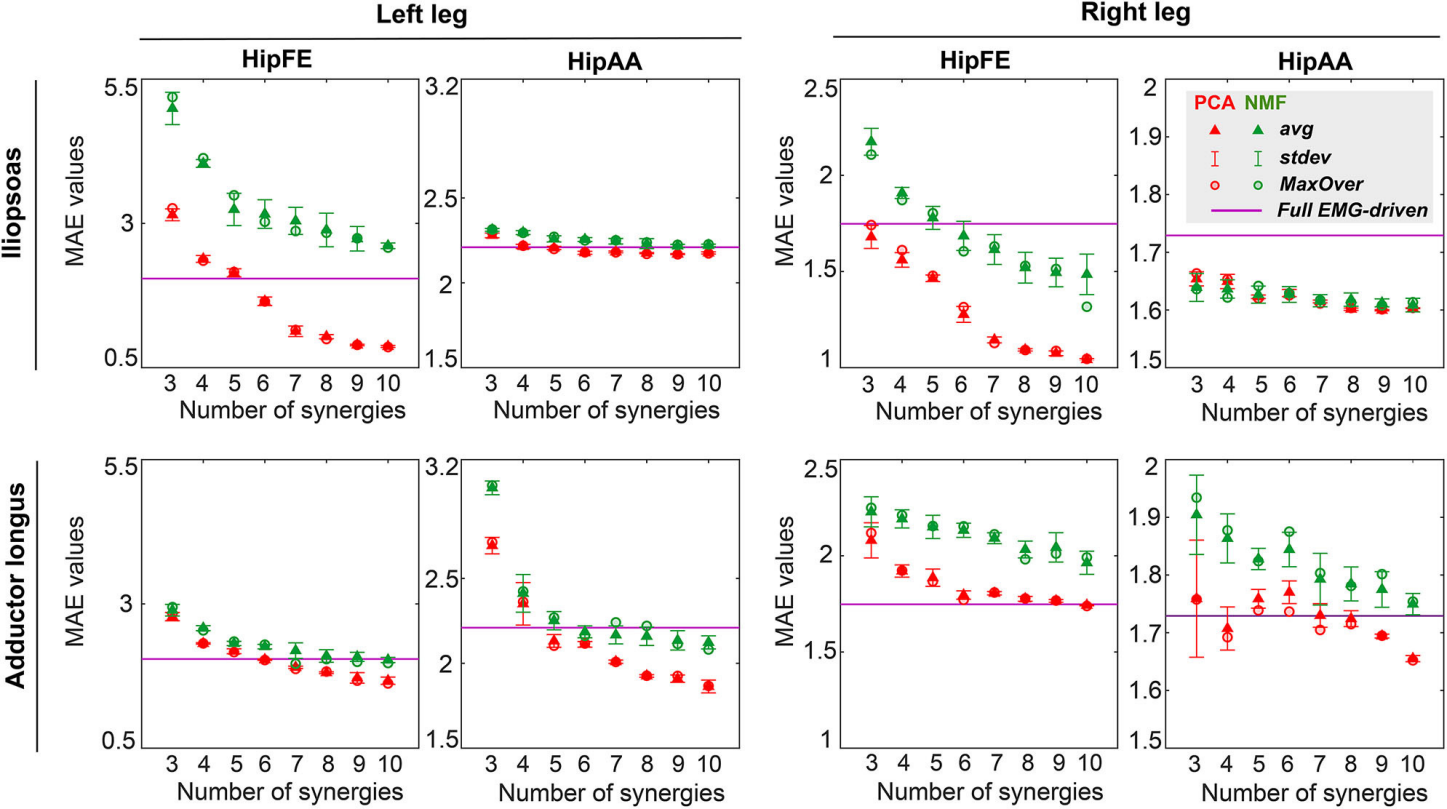
Average (triangles) and standard deviation of mean absolute error (MAE) values for hip joint moment prediction across all trials (including both calibration and evaluation) and all EMG normalization methods as a function of the number of synergies for both legs (left leg: non-paretic, right leg: paretic). MAE values for joint moment prediction are presented for hip flexion/extension (HipFE) and hip adduction/abduction (HipAA). PCA- and NMF-based SynX results are indicated in red and green, respectively. The flat purple lines demonstrate the average MAE values for joint moment prediction with a full set of EMG signals for each leg. Red circular (PCA) and green circular (NMF) markers show average MAE values across all trials using MaxOver normalization.

The three-factor ANOVA revealed that MAE values for joint moment matching were sensitive to both matrix factorization algorithm and number of synergies (*p* < 0.01) but insensitive to EMG normalization method (*p* = 0.12) for all muscle-leg-joint combinations. Furthermore, no statistically significant interaction effects were observed among the three factors for MAE values. For the HipFE moment, MAE values for both PCA-based and NMF-based SynX decreased as the number of synergies increased (*p* < 0.01). Nonetheless, PCA produced more accurate joint moment matching, as indicated by smaller MAE values, than did NMF (*p* < 0.01). In contrast, for the HipAA moment, neither number of synergies nor matrix factorization algorithm had a significant influence on MAE values. The one exception was adductor longus in the left leg, where MAE values decreased with an increasing number of synergies for both algorithms, though PCA had significantly smaller MAE values than did NMF (*p* < 0.01) (Figure 6 and Supplementary Figure 3).

PCA-based and NMF-based SynX led to different levels of joint moment tracking accuracy compared to the full EMG-driven model calibration (Figure 6 and Supplementary Figures 3, 4). For example, for the HipFE moment with MaxOver normalization, the average MAE values for PCA-based SynX dropped below the average MAE from the full EMG-driven calibration at six synergies for iliopsoas in the left leg, six synergies for adductor longus in the left leg, and ten synergies for adductor longus in the right leg. In contrast, for the HipFE moment with MaxOver normalization, the MAE values for NMF-based SynX dropped below the average MAE from the full EMG-driven model calibration at six synergies for iliopsoas in the right leg and seven synergies for adductor longus in the left leg.

**Figure 7:**
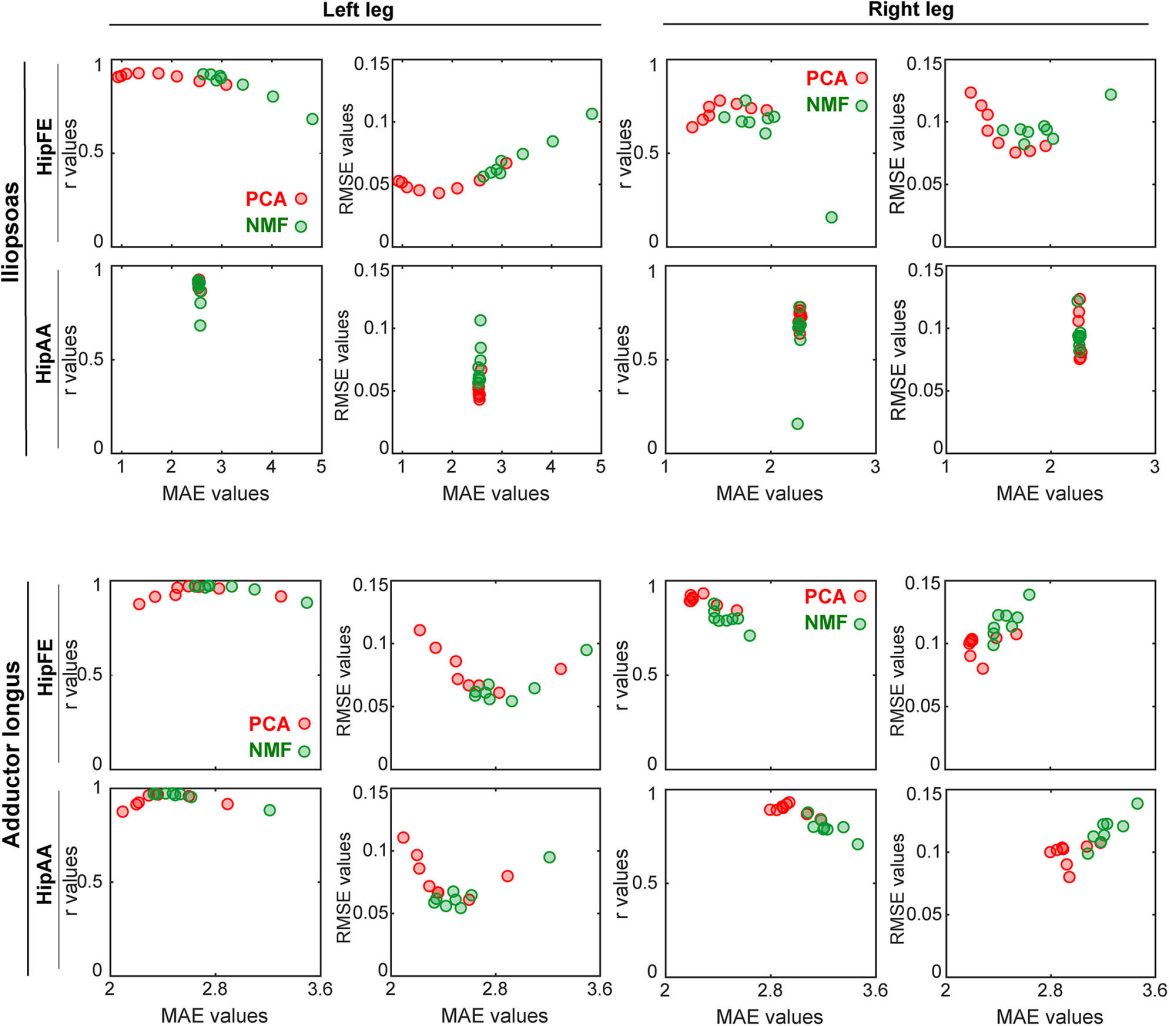
Trade-offs between accuracy of joint moment tracking (MAE values on horizontal axis) and accuracy of unmeasured muscle excitation reconstruction (*r* or RMSE values on vertical axis) when using MaxOver as the EMG normalization method (red markers: PCA-based SynX; green markers: NMF-based SynX; HipFE: hip flexion/extension; HipAA: hip adduction/abduction). The left leg is non-paretic and the right leg is paretic.

When *r* and RMSE values for muscle excitation matching were plotted as a function of MAE values for joint moment matching (Figure 7 and Supplementary Figure 5), the observed trends were different for PCA- vs. NMF-based SynX. Taking MaxOver normalization as an example in Figure 7, for PCA-based SynX, the trends were parabolic, with a small region of MAE values corresponding to both largest *r* values and smallest RMSE values. In contrast, for NMF-based SynX, the observed trends were approximately linear, with the smallest MAE errors corresponding to the largest *r* values and smallest RMSE values. For both SynX methods, MAE values for joint moment matching were approximately the same in the region where *r* values were the largest and RMSE values the smallest.

## Discussion

This study demonstrated that SynX is able to predict unmeasured muscle excitations with reasonable reliability using a well-calibrated EMG-driven model. However, the reliability of the predictions was heavily influenced by the matrix factorization algorithm used and number of synergies, while EMG normalization method had little influence on SynX results. Our results clearly showed that PCA was able to generate estimates of unmeasured muscle excitations that were more accurate in shape and magnitude, more robust to EMG normalization, and more consistent across all unmeasured muscle-leg combinations in comparison with NMF. The results also highlight that for PCA-based SynX, a relatively low number of synergies, typically five or six, always provided the most accurate predictions of unmeasured muscle excitations and joint moments simultaneously, while NMF was either unable to reproduce unmeasured muscle excitations with reasonable accuracy or required a large number of synergies, typically above eight, to attain comparable results to those of PCA.

The SynX method optimizes unmeasured synergy vector weights by tracking joint moments as closely as possible. We used the muscle synergy concept for the prediction of missing muscle excitations for several reasons. First, it has been theorized that muscle synergies are generated by the central nervous system to regulate the control of highly redundant musculoskeletal systems in an efficient manner (Tresch et al., 2006; Torres-Oviedo and Ting, 2007). Second, the problem of finding unknown time-varying muscle excitations is reduced to the problem of finding a small number of synergy vector weights associated with unmeasured muscles, which significantly decreases the search space for the optimization in comparison with SO-based approaches. Third, there are no abrupt changes in predicted muscle excitations as is often observed with SO-based approaches, since predicted muscle excitations are linear combinations of weighted synergy excitations that are normally smooth. Fourth, by finding unknown synergy vector weights within the context of an EMG-driven modeling framework, the method is practically applicable in contrast to previous work that only demonstrated theoretical feasibility (Bianco et al., 2018). Fifth, SynX results demonstrated that joint moments were matched as accurately as could be achieved by the full EMG-driven model (Supplementary Figure 3) (Meyer et al., 2017). Given these observations, the proposed SynX method may outperform other EMG-driven muscle force estimators that either ignore deep muscles or use SO-based methods to estimate EMG signals for inaccessible or unavailable muscles.

For the same number of synergies, PCA generally predicted unmeasured muscle excitations with greater accuracy than did NMF (Figure 4). PCA and NMF are two of the most popular matrix factorization algorithms used for performing MSA. Although the MSA literature suggests that NMF generates synergy components that are highly correlated with those generated by PCA (Ivanenko et al., 2005; Cappellini et al., 2006; Banks et al., 2017), each of the algorithms decomposes a given data set with different assumptions and constraints (Ting and Chvatal, 2010; Gallina et al., 2018). PCA is based on linear algebra and maintains orthogonality among all principal components during factorization, while NMF is based on nonlinear optimization with potentially non-unique solutions in the non-negative space (Supplementary Figure 1). PCA has been rejected by a few studies due to the inherent non-negative nature of muscle excitations (Ajiboye and Weir, 2009; Banks et al., 2017). However, our findings demonstrated several benefits of PCA over NMF for SynX purposes.

At least three observations help explain why PCA-based SynX generally worked better than did NMF-based SynX. First, for an equal number of synergies, PCA-derived components accounted for more variance in measured muscle excitations than did those derived using NMF, for three to five synergies in particular (Table 2). Our findings agree well with the results of variance accounted for by different numbers of synergies when PCA and NMF are applied to high-density EMG data (Gallina et al., 2018). Second, PCA identifies synergy components that tend to describe the direction of the largest variance in the measured muscle excitations, with subsequent components being perpendicular to the previous ones, while NMF finds components that tend to represent the edge of a convex subspace in which all original EMG measurements lie (Ting and Chvatal, 2010; Lambert-Shirzad and Van der Loos, 2017). Furthermore, any of the data in the reduced-dimensionality space could be reconstructed due to the negative and positive weights allowed by PCA. However, only data points representing unmeasured muscle excitations that were located within the defined subspace could be closely reproduced by NMF due to the non-negatively constraint on the solutions (Tresch et al., 2006; Ting and Chvatal, 2010). Third, PCA-based SynX had one more design variable—an offset - for each trial that represents the average value of the unmeasured muscle excitation, thereby adding one extra degree of freedom to the optimization problem. Therefore, better performance of PCA-based than NMF-based SynX may be due to the non-negativity constraints for NMF and the extra design variables for PCA, both of which make the feasible search space of NMF more restricted than that of PCA.

SynX performance was heavily influenced by the number of synergies used. Previous studies have reported that three to six synergies are adequate for reconstructing measured EMG signals with over 90% variance accounted for during gait (Ivanenko et al., 2005; Torres-Oviedo and Ting, 2007; Clark et al., 2009; Banks et al., 2017; Bianco et al., 2018), consistent with the observations in this study (Table 2). Not surprisingly, as the number of synergies increased, reconstruction accuracy of unmeasured muscle excitations using NMF increased before reaching a plateau, which was similar to reconstruction behavior for measured muscle excitations (Table 2). However, PCA-based SynX performance with an increasing number of synergies exhibited non-monotonic behavior (Figure 4 and Supplementary Figure 2). For both NMF- and PCA-based SynX, as the number of synergies increased, the additional degrees of freedom in the optimization allowed the optimizer to achieve lower joint moment tracking errors (Figure 6 and Supplementary Figure 4). When the number of synergies was above a certain number, the joint moment tracking errors achieved by PCA-based SynX dropped below those achieved by the full EMG-driven optimization, with the prediction of unmeasured muscle excitations becoming less accurate as a consequence.

Another important observation was that as the number of synergies increased, the best SynX results were generated for the number of synergies at which joint moment tracking errors became lower than those produced by the full EMG-driven model. PCA-based SynX could generate joint moment tracking errors that were comparable to those for the full EMG-driven calibration using a relatively smaller number of synergies, whereas NMF-based SynX could not. Due to the non-negativity constraints built into NMF-based SynX, joint moment tracking errors for that method sometimes (e.g., iliopsoas in the left leg for HipFE) stayed above or around the levels achieved by the full EMG-driven model, which caused SynX performance using NMF to level off. Therefore, even though the number of synergies that generated the best PCA-based SynX results varied across trials, muscles, and legs, PCA always needed fewer synergies than did NMF. This fact simplifies the implementation of our method and may improve computational efficiency when more variables need to be calibrated simultaneously.

Though EMG normalization methods had no statistically significant influence on SynX performance using PCA and NMF, the variability of average *r* and RMSE values across all five EMG normalization methods was considerably less for PCA than that for NMF using the same number of synergies (Figure 4 and Supplementary Figure 2). PCA generates principal components that successively maximize variance (Tresch et al., 2006), which explains why each PCA-derived synergy excitation representing the corresponding variance for each principal component exhibited significant magnitude reduction (Supplementary Figure 1). In addition, the low-dimensional space in PCA defined by orthogonal principal components rotates as data are scaled differently across different muscles, but the rotated principal components are still highly descriptive of any points in the low-dimensional orthogonal space (Ting and Chvatal, 2010). In contrast, NMF decomposition is based on assessing the quality of magnitude approximation, and scaling of original data differently across muscles would lead to deformation of the convex subspace that the components surround. With certain scaling schemes or normalization methods, the points accounting for unmeasured muscle excitations may not remain within the newly-generated NMF subspace. Therefore, NMF-based SynX performance would be sensitive to EMG normalization method, which is in agreement with the conclusions for NMF-based decomposition reported by Tresch et al. (2006). For different EMG normalization methods, the optimizer is presented with a more consistent search space for PCA than for NMF, which could explain why PCA is less sensitive to EMG normalization method than is NMF. Therefore, PCA may eliminate the need to identify the true maximum muscle excitation for EMG normalization purposes in preparation for SynX, which is a significant advantage over NMF.

In general, for a given number of synergies, the non-paretic side (left leg) had higher reconstruction quality for both measured and unmeasured muscle excitations than did the paretic side (right leg) (Table 2). This observation is inconsistent with the findings reported by Clark et al. (2009) but consistent with previous synergy analysis of the same data set (Bianco et al., 2018). The underlying reason for this discrepancy could be the methodological differences between Clark et al.'s and our study. For example, we performed MSA on a different set of muscles, which may have influenced the MSA results (Steele et al., 2013; Banks et al., 2017). Furthermore, the single subject post-stroke who participated in our study was high-functioning whereas Clark et al. studied 55 subjects post-stroke with heterogeneous characteristics. Interestingly, our study found that reconstruction of unmeasured muscle excitations through SynX was more accurate for the non-paretic side (left leg) than for the paretic side (right leg) (Figure 4), which is possibly due to higher amplitude EMG signals for the paretic leg. For instance, predicted adductor longus peaks during swing phase (60–100% of gait cycle) were larger for the paretic side, and SynX was unable to reproduce them as accurately as for the non-paretic side (Figures 2, 3). In addition, to interpret the model calibration difference between legs, we observed that for the non-paretic side (left leg), as the number of synergies increased, the joint moment tracking error for PCA-based SynX started above and then dropped below the average from the full EMG-driven model calibration (Figure 6). However, this behavior was not observed for the paretic side (right leg), and consequently no number of synergies could generate muscle excitations and joint moments together as accurately as with the full EMG-driven model calibration.

Our study involved several important limitations that suggest areas for future investigation. First, we calibrated the EMG-driven model with a full set of EMG signals to obtain personalized model parameters (e.g., EMG scale factors, electromechanical delays, musculotendon parameter values, and geometric coefficients), and SynX worked well when the calibrated model parameters were held constant. In the future, a challenge will be to develop an optimization problem formulation where design variables defining both unmeasured synergy vector weights and EMG-driven model parameters are found simultaneously while still predicting joint moments and unmeasured muscle excitations accurately. Second, the extraction of measured muscle synergies was performed before the optimization process. However, since changing some parameter values (e.g., EMG normalization factors) can change the synergy analysis outcome, SynX may work better if MSA was performed iteratively within the optimization. Since PCA leads to unique and fast factorization solutions for synergy excitations while NMF does not (Tresch et al., 2006), PCA would be the preferred decomposition method for such an iterative approach. Third, the SynX framework was validated on cases where only one hip muscle (e.g., iliopsoas or adductor longus) was assumed to be missing at a time. The approach should be evaluated more extensively on cases where multiple muscles are assumed to be unmeasured concurrently. Fourth, unmeasured synergy vector weights were allowed to vary between trials in the current study. It would be worthwhile to explore whether making synergy vector weights subject-specific or task-specific can lead to simplification of the SynX algorithm or improvements in SynX performance. Lastly, this study used gait data from only a single subject post-stroke with extensive EMG data. SynX needs to be investigated in diverse subject populations, dynamic movement conditions, and experimental scenarios with other unmeasured muscles.

## Conclusion

This study showed that SynX is a viable option for estimating an unmeasured muscle excitation using synergy excitations extracted from measured muscle excitations. The study also demonstrated that methodological choices (i.e., matrix factorization algorithm and number of synergies) made before MSA affect the accuracy with which unmeasured muscle excitations can be predicted. The synergy vector weights associated with an unmeasured muscle excitation were optimized by minimizing errors between the EMG-driven model predicted and experimental joint moments. Our results highlighted that PCA was able to provide more accurate, reliable, and efficient estimates of unmeasured muscle excitations than was NMF. In general, PCA required five or six synergies to achieve the best prediction of unmeasured muscle excitations, and inclusion of additional synergies reduced SynX performance. Better SynX performance for PCA may be the result of the non-negativity restrictions imposed by NMF. Moreover, PCA was less sensitive to EMG normalization method than was NMF, which may reduce the need to identify the true maximum muscle excitations reliably for EMG normalization. SynX could be useful to address difficulties in collecting EMG signals from deep muscles inaccessible by surface electrodes, which is critical when predicting muscle forces with an EMG-driven musculoskeletal model. It could also be useful when using EMG systems with fewer channels than desired. Our proposed SynX method may eventually facilitate the assessment of human neuromuscular control and biomechanics after rehabilitation or surgical treatment when EMG data collection is limited.

## Data Availability Statement

The experimental data, OpenSim model, and Matlab code used to perform this study are available at https://simtk.org/projects/synx.

## Ethics Statement

All experimental procedures were approved by the University of Florida Health Science Center Institutional Review Board (IRB-01), and the subject provided written informed consent prior to participation.

## Author Contributions

CP and BF designed and performed the experiments. DA analyzed the data, prepared the figures, and drafted the manuscript. MS and BF supervised the research. DA, MS, CP, and BF revised the manuscript and approved the final version.

## Conflict of Interest

The authors declare that the research was conducted in the absence of any commercial or financial relationships that could be construed as a potential conflict of interest.
