# Supplementary material for: Evaluation of Synergy Extrapolation for Predicting Unmeasured Muscle Excitations from Measured Muscle Synergies
Source: Front Comput Neurosci. 2020 Dec 4;14:588943. doi: 10.3389/fncom.2020.588943 (PMC7746870; doi:10.3389/fncom.2020.588943)
Supplement: Supplementary file 1 [file Data_Sheet_1.PDF]

Supplementary Material

1 Muscles in each leg

Table S1 List of the muscles in the EMG-driven model and corresponding degrees of freedom (DOFs).

EMG channel	EMG label	EMG type	DOFs	Muscle
Adductor longus	AddLong	Fine wire	Hip FE/Hip AA	Adductor brevis
				Adductor longus
				Adductor magnus distal
				Adductor magnus ischial
				Adductor magnus middle
				Adductor magnus proximal
Gluteus maximus	GlutMax	Surface		Gluteus maximus superior
				Gluteus maximus middle
				Gluteus maximus inferior
Gluteus medius	GlutMed	Surface		Gluteus medius anterior
				Gluteus medius middle
				Gluteus medius posterior
				Gluteus minimus anterior
				Gluteus minimus middle
				Gluteus minimus posterior
Iliacus or Psoas	Iliopsoas	Fine wire		Iliacus
				Psoas
Semimembranosus	SemiMemb	Surface	Hip FE/Hip AA/Knee FE	Semimembranosus
				Semitendinosus
Biceps femoris long head	BicFemLong	Surface	Hip FE/Hip AA/Knee FE	Biceps femoris long head
			Knee FE	Biceps femoris short head
Rectus femoris	RecFem	Surface	Hip FE/Hip AA/Knee FE	Rectus femoris
Vastus medialis	VastMed	Surface	Knee FE	Vastus medialis
				Vastus intermedius
Vastus lateralis	VastLat	Surface		Vastus lateralis
Medial gastrocnemius	GasMed	Surface	Knee FE/Ankle PDF/Ankle IE	Lateral gastrocnemius
				Medial gastrocnemius
Tibialis anterior	TibAnt	Surface	Ankle PDF/Ankle IE	Tibialis anterior
Tibialis posterior	TibPost	Fine wire		Tibialis posterior
Peroneus longus	PeroneusLong	Surface		Peroneus brevis
				Peroneus longus
				Peroneus tertius
Soleus	Sol	Surface		Soleus
Extensor digitorum longus	ExtDigLong	Fine wire		Extensor digitorum longus
Flexor digitorum Longus	FlexDigLong	Fine wire		Flexor digitorum Longus

Hip FE: hip flexion/extension; Hip AA: hip adduction/abduction; Knee FE: knee flexion/extension; Ankle PDF: ankle plantarflexion/dorsiflexion; Ankle IE: ankle inversion/eversion.

2 Muscle synergy analysis

Representative synergy components identified by PCA (Figure 1 (A)) composed of synergy excitations and corresponding weights that were both positive and negative. The percentage of variance that each synergy component explained decreased from 64% to 1% from the first to the sixth synergy, where the magnitude of each corresponding synergy excitation also experienced a significant drop. In contrast to PCA, the synergy excitations and weights determined by NMF (Figure 1 (B)) contained only non-negative values as constrained by the algorithm itself, and the variance that each component accounted for stayed at comparable levels. All the NMF-identified synergy excitations also had comparable magnitudes.

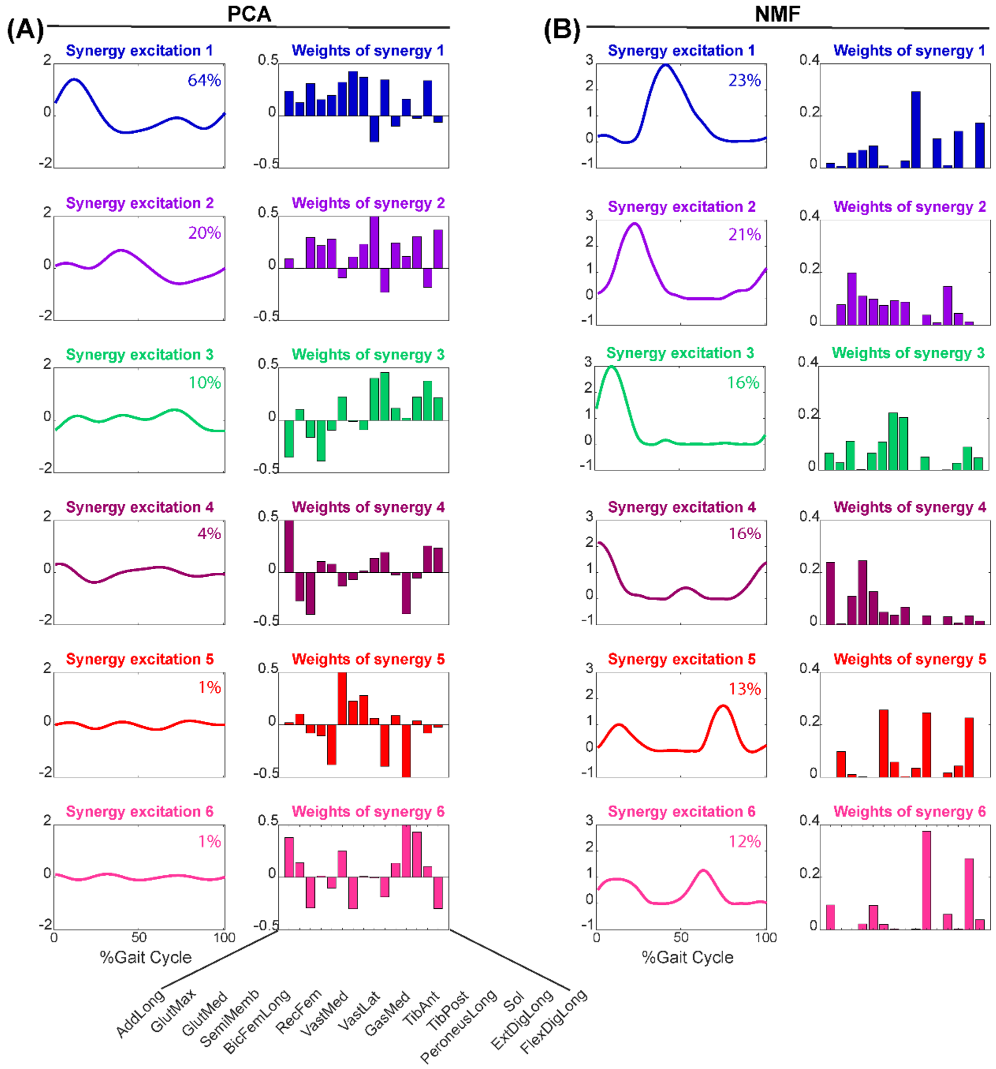

Fig. S1 Representative results of synergy excitations and corresponding synergy weights from measured EMGs identified using (A) PCA and (B) NMF, respectively. The sample data set was collected from the non-paretic side (left) of the patient when walking at his self-selected speed (0.5 m/s). 'Iliopsoas' EMG channel was assumed to be unmeasured, and the remaining 15 EMG channels were normalized to maximum values over all trials (MaxOver) before being decomposed for this case. Percentages represent the amount of total data VAF contributed by each synergy excitation, and the synergy components were sorted according to the descending percentage of VAF.

3. Synergy extrapolation performance

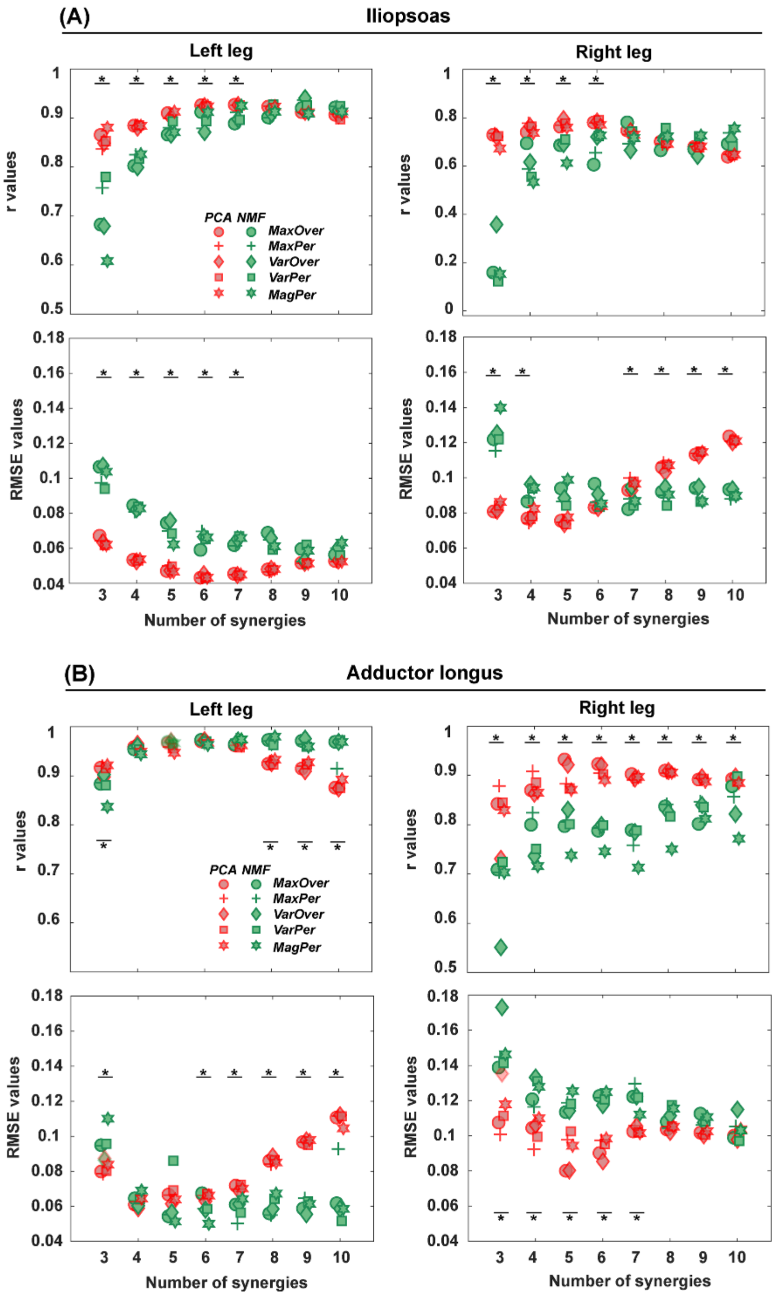

Fig. S2 Average r and RMSE values for the reconstruction of iliopsoas (A) and adductor longus (B) muscle excitations across all trials (including both calibration trials and evaluation trials) for both legs (right leg: paretic, left leg: non-paretic) with 3 to 10 synergies (red markers: PCA-based synergy extrapolation; green markers: NMF-based synergy extrapolation; 5 EMG normalization methods are represented by different marker shapes; black bars represent a significant difference between PCA and NMF when matched for the number of synergies, $p \leq 0.05$).

4. Joint moment prediction

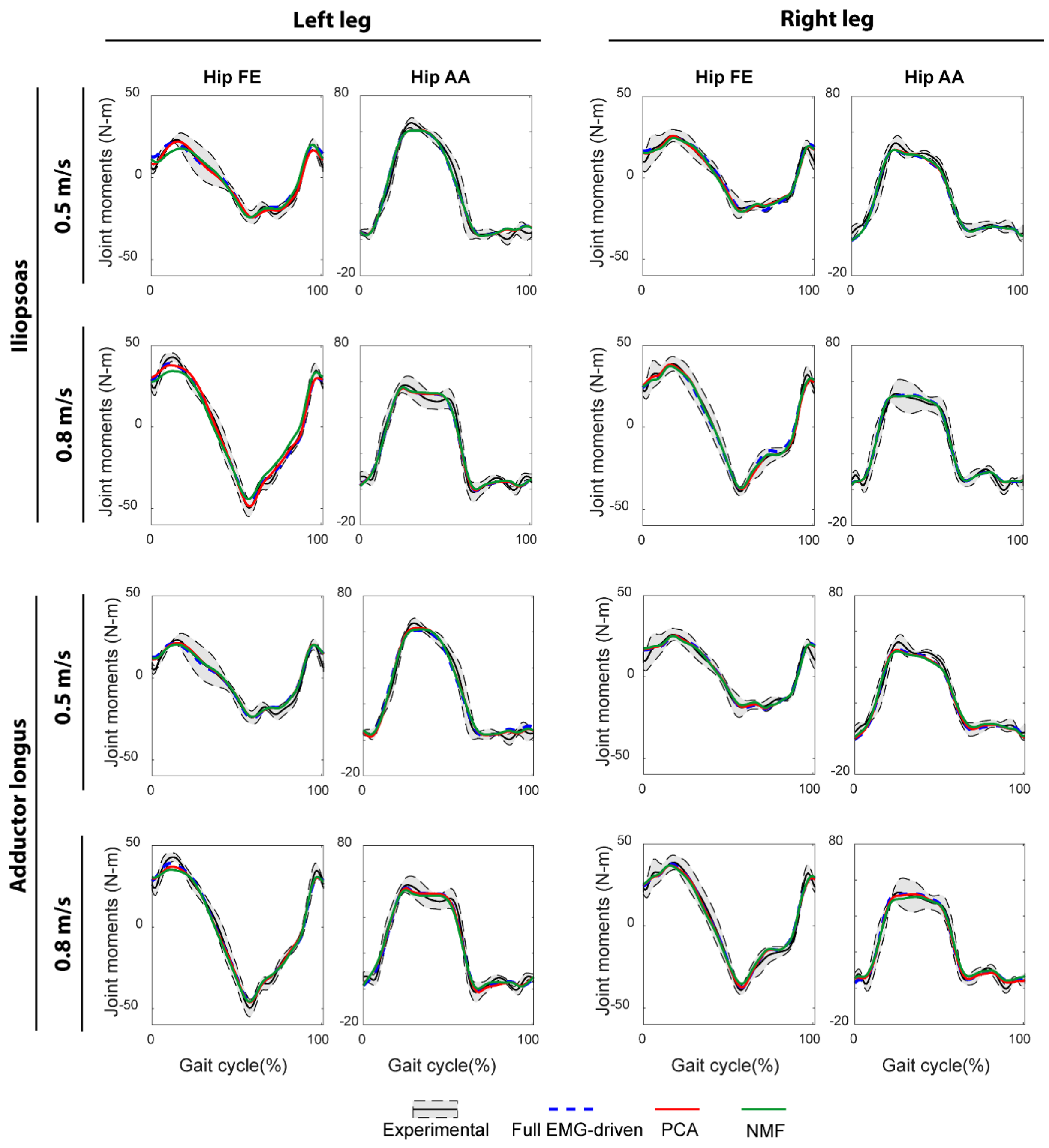

Fig. S3 Average joint moment prediction across all calibration trials when performing full EMG-driven model calibration on step 1 (blue dash line) and synergy extrapolation on step 2 with PCA (red line) or NMF (green line), respectively. Black lines stand for the experimental joint moments from inverse dynamics, and the grey shaded areas represent ± 1 standard deviation. Six synergies are used for the generation of these representative results. Data is reported for the whole gait cycle with 0% being heel-strike and 100% being consecutive heel-strike events for both legs (right leg: paretic, left leg: non-paretic).

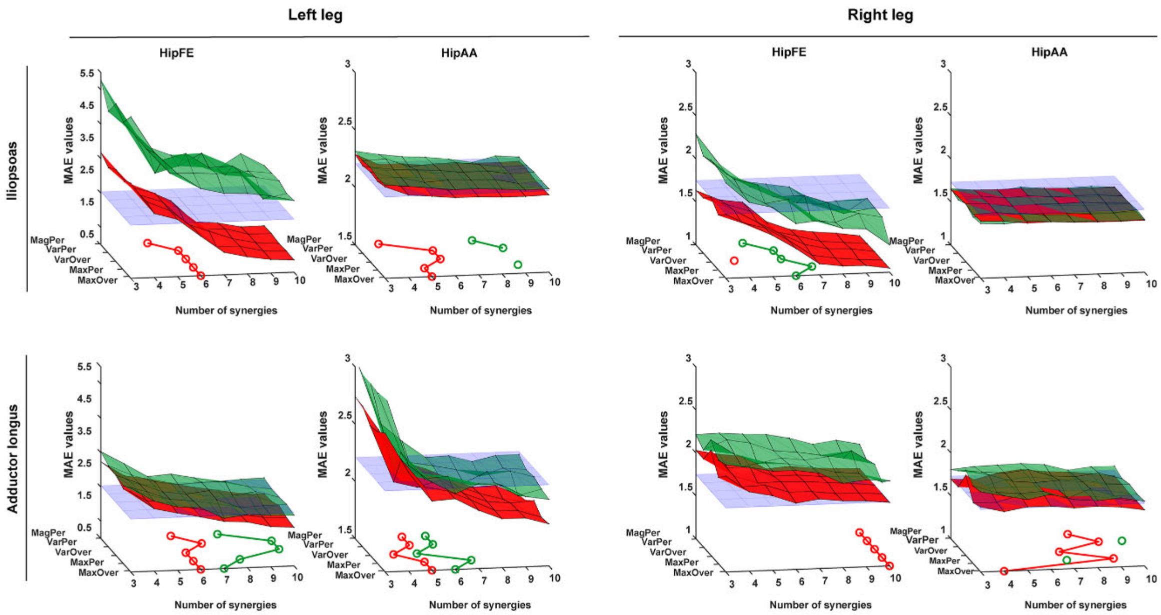

Fig. S4 3D plots report the average MAE values for hip joint moment prediction across all trials by using 6 EMG normalization methods with an increase of the number of synergies from 3 to 10. The MAE values are averaged across all calibration and evaluation trials at 2 walking speeds for both legs (right leg: paretic, left leg: non-paretic). PCA- and NMF-based synergy extrapolations are indicated in red and green surfaces, respectively. The flat purple planes demonstrate the average MAE values for joint moment prediction with a full set of EMG signals at a specific leg. The circle lines on the bottom walls indicate where 3D surfaces of average MAE values for PCA- and NMF-based synergy extrapolation first intersect with the plane of average MAE for the full EMG-driven calibration, respectively, as the number of synergies increases. The absence of a circle means that there was no intersection corresponding to that synergy.

5. Joint moment prediction vs synergy extrapolation performance

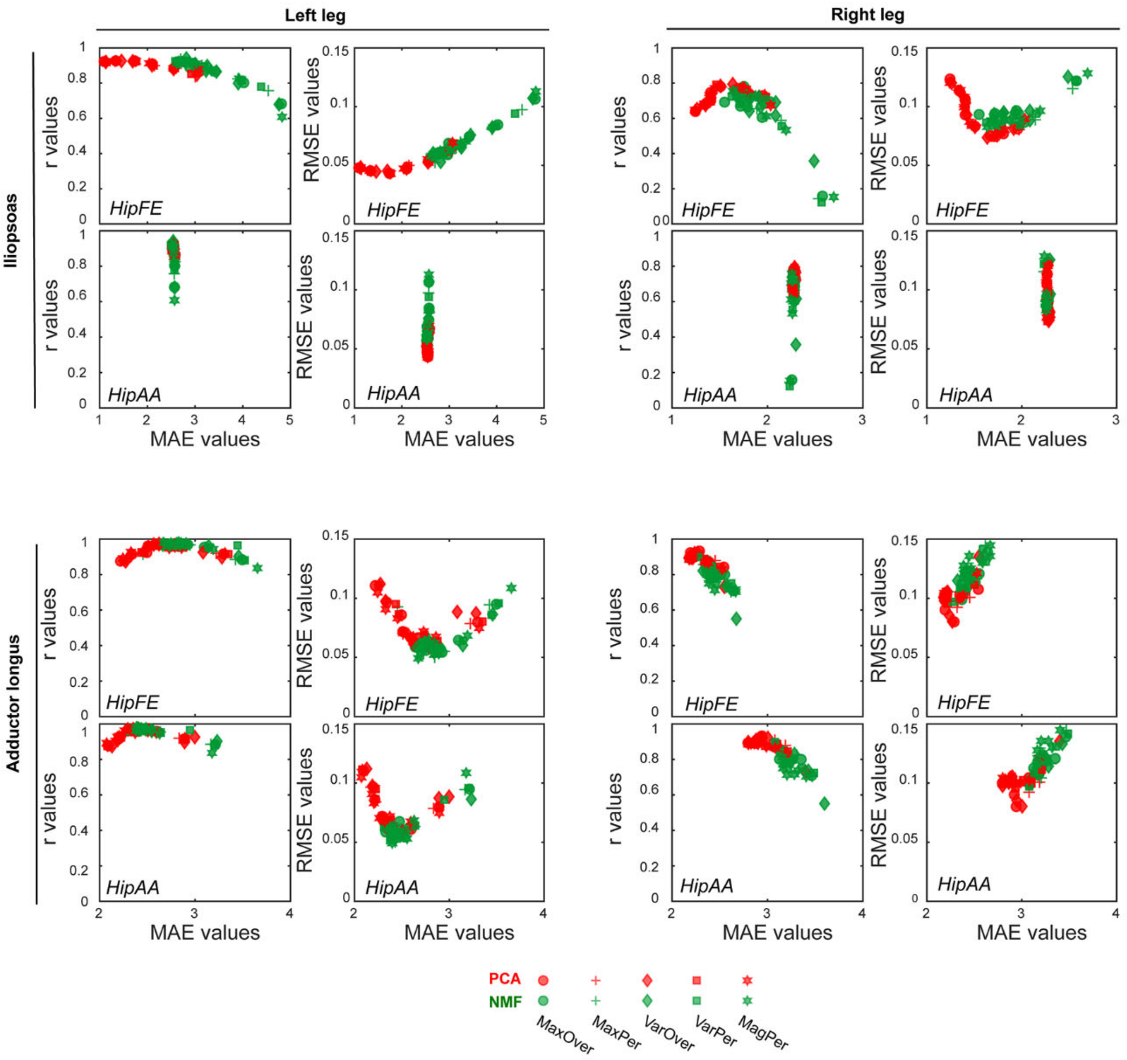

Fig. S5 The trade-off relationship between the accuracy of joint moment tracking (indicated by MAE values in the horizontal axis) and accuracy of unmeasured muscle excitation reconstruction (indicated by r values or RMSE values in the y-axis) (red markers: PCA-based synergy extrapolation; green markers: NMF-based synergy extrapolation; 5 EMG normalization methods are represented by different marker shapes; the right leg is paretic, and the left leg is non-paretic).
